# Automated Lung Ultrasound Pulmonary Disease Quantification Using an Unsupervised Machine Learning Technique for COVID-19

**DOI:** 10.3390/diagnostics13162692

**Published:** 2023-08-16

**Authors:** Hersh Sagreiya, Michael A. Jacobs, Alireza Akhbardeh

**Affiliations:** 1 Department of Radiology, Perelman School of Medicine, University of Pennsylvania, Philadelphia, PA 19104, USA; 2 The Russell H. Morgan Department of Radiology and Radiological Science, The Johns Hopkins University School of Medicine, Baltimore, MD 21205, USA; 3 Department of Diagnostic and Interventional Imaging, The University of Texas Health Science Center, Houston, TX 77030, USA; 4 Ambient Digital LLC, Daly City, CA 94014, USA

**Keywords:** ultrasound, machine learning, COVID-19, unsupervised learning, POCUS, computer vision, treatment effectiveness

## Abstract

COVID-19 is an ongoing global health pandemic. Although COVID-19 can be diagnosed with various tests such as PCR, these tests do not establish pulmonary disease burden. Whereas point-of-care lung ultrasound (POCUS) can directly assess the severity of characteristic pulmonary findings of COVID-19, the advantage of using US is that it is inexpensive, portable, and widely available for use in many clinical settings. For automated assessment of pulmonary findings, we have developed an unsupervised learning technique termed the calculated lung ultrasound (CLU) index. The CLU can quantify various types of lung findings, such as A or B lines, consolidations, and pleural effusions, and it uses these findings to calculate a CLU index score, which is a quantitative measure of pulmonary disease burden. This is accomplished using an unsupervised, patient-specific approach that does not require training on a large dataset. The CLU was tested on 52 lung ultrasound examinations from several institutions. CLU demonstrated excellent concordance with radiologist findings in different pulmonary disease states. Given the global nature of COVID-19, the CLU would be useful for sonographers and physicians in resource-strapped areas with limited ultrasound training and diagnostic capacities for more accurate assessment of pulmonary status.

## 1. Introduction

SARS-CoV-2, also known as COVID-19, is a global pandemic that has led to over six hundred million documented cases and 6.8 million deaths, and it has resulted in devastating economic damage as of early 2023 [1]. Radiological imaging is useful for assessing pulmonary disease burden, assessing disease severity, and tracking the disease course [2]. Although X-rays are both portable and inexpensive, they lack sensitivity compared to CT, with a reported sensitivity of 69% [3]. While CT provides excellent anatomic imaging, its use in the United States is typically limited to acute cases, it is not portable, and it may be difficult to access for longitudinal monitoring due to logistical concerns, cost, and cumulative radiation dose [4,5]. However, ultrasound (US) is relatively inexpensive, portable, and widely available, and it has successfully been used to monitor lung diseases [6]. In fact, recent studies have shown lung ultrasound findings to demonstrate high diagnostic sensitivity and accuracy, comparable to CT [7]. Due to its portability, ultrasound can be taken directly into a patient’s room, and modern ultrasound scanning devices, such as Butterfly iQ, can be used as point-of-care devices connected to a smartphone [8]. Consequently, many emergency departments (ED) have made ultrasound a mainstay for early COVID-19 diagnosis in patients presenting with flu-like symptoms [9]. For instance, the CLUE (COVID-19 lung ultrasound in the ED) protocol includes a lung ultrasound scoring system (LUSS) for rating the severity of pulmonary findings [10]. LUSS has previously shown utility for COVID-19 and other respiratory illnesses [11]. Several authors have proposed scoring systems for lung ultrasound in COVID-19 [12,13,14].

Various pulmonary findings are evident on ultrasound, including A-lines, B-lines, consolidations, and pleural effusions, with characteristic lung ultrasound findings associated with COVID-19 [15]. Ultrasound can be performed at the patient’s bedside, whether in an ED, inpatient floor, intensive care unit, or field hospital. Its inexpensive cost and deployable nature dramatically increase US potential for worldwide availability. Nevertheless, ultrasound does have potential challenges. First, it is operator-dependent, relying on the skills of the sonographer. Second, lung US has not traditionally been performed as frequently as other forms of ultrasound, such as abdominal, pelvic, and obstetric ultrasound.

Although sonographers have become better versed with lung ultrasound during the COVID-19 pandemic, there still exists a potential problem regarding quantifying the different US lung findings. To address this problem, we have developed a technique that uses an unsupervised learning technique, a patient-specific model, that does not require a large patient cohort, which is typically required for successfully training supervised artificial intelligence (AI) algorithms.

**Figure 1:**
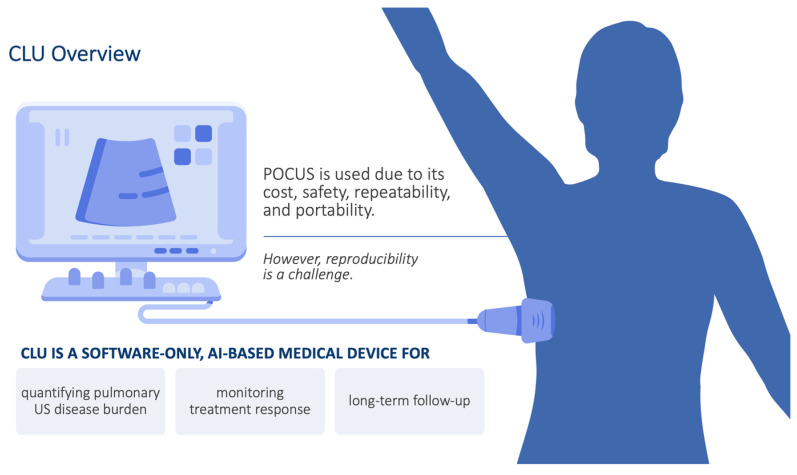
Overview of the CLU technology. POCUS = point-of-care ultrasound.

Although COVID-19 is diagnosed with PCR, this test does not establish the extent of disease within the lungs, only the presence of the virus. This is crucial since pulmonary involvement is important for determining disease severity and response to treatment. There are now several treatments for COVID-19, such as nirmatrelvir-ritonavir, a combination of oral protease inhibitors that are used in symptomatic outpatients at risk for progression to severe disease [16]. Patients under treatment will need to be monitored longitudinally to ensure that their pulmonary disease burden is improving. We have developed a technique termed the calculated lung ultrasound (CLU) that quantifies several imaging characteristics (Figure 1). CLU can be used to estimate the extent of lung involvement, especially important for the initial staging of COVID-19, as well as the longitudinal disease course, and it provides a method to evaluate if treatment is working. When used in conjunction with clinical data, the information about pulmonary disease burden could help guide patient management by highlighting specific lung tissue characteristics, such as A or B-lines, pleural effusions, and consolidation.

The objectives of this study were to describe the CLU technique and evaluate its performance in identifying key lung ultrasound findings related to COVID-19 on an initial dataset of 52 ultrasound examinations.

## 2. Materials and Methods

### 2.1. Clinical Information

This is a retrospective study involving several institutions and public databases. The multi-institutional retrospective data used in this paper were read by board-certified physicians. All data were deidentified. There were 52 ultrasound examinations scanned with multiple US scanners including Siemens, Philips, GE, Butterfly iQ, and SonoSite. For lung ultrasound imaging, curvilinear or phased-array 5–9 MHz probes with a small convex tip are typically used to examine the lung, as they can easily be placed in the intercostal space. However, a linear probe with a higher frequency (6–13 MHz) can be used to assess soft tissues, ribs, lung sliding, and the pleura. On the other hand, a convex probe with a low frequency (3–5 MHz) can be used to assess depth for effusions, consolidations, and extension of B-lines [17].

Ultrasound findings were verified by using the reports of radiologists with expertise in identifying lung ultrasound findings associated with COVID-19.

### 2.2. Lung Ultrasound Features

**Figure 2:**
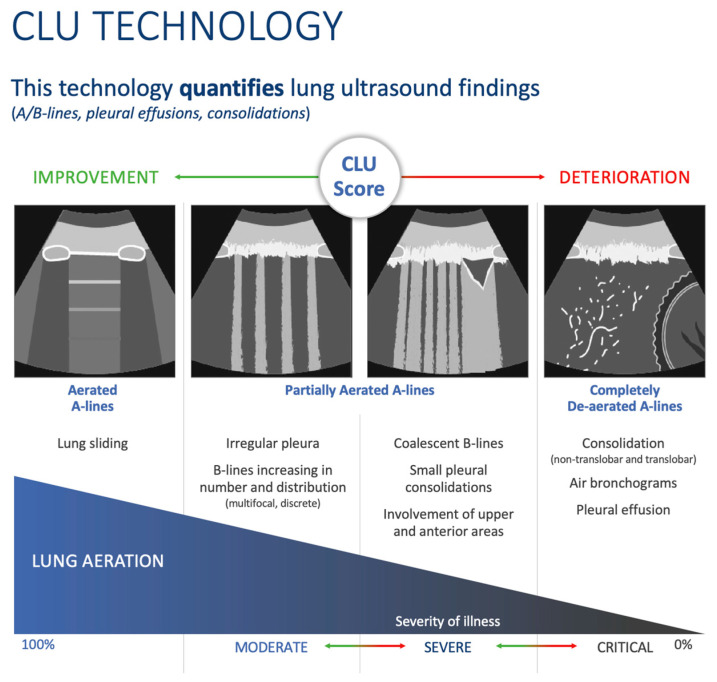
Sonographic characteristics of moderate, severe, and critical changes in patients with COVID-19.

Lung US assesses different artifacts termed A-lines and B-lines. A-lines are horizontal lines that represent normal aerated lungs (dry interlobular septa), representing a reverberation artifact caused by sound waves bouncing off highly echogenic pleura and back to the probe [18]. B-lines represent the correlate of Kerley B-lines on chest radiograph; they are vertical lines of hyperechoic artifact, originating from water-thickened pulmonary interlobular septa; they have been compared to the beam of a flashlight and are commonly seen in lungs with interstitial edema. They originate at the pleural line and traverse the entire ultrasound screen vertically to the bottom of the screen. Potential causes of unilateral B-lines include pneumonia and pulmonary contusion. For an ultrasound exam to be deemed positive, there are typically greater than three B-lines per view [19]. C-lines are seen when there is an area of echogenicity arising below a subpleural consolidation [20]. Figure 2 illustrates examples of various ultrasound findings evaluated by CLU.

### 2.3. Calculated Lung Ultrasound Algorithm

The CLU, which was developed using MATLAB, is outlined below:

1. Computer Vision and Image Segmentation: This component of CLU uses image segmentation and video processing techniques, which include clustering methods and non-linear manifold learning, to detect lung features used by radiologists on lung ultrasound exams [21,22,23,24]. These features were described in Figure 2 and Section 2.2, including A-lines, B-lines, consolidation, pleural effusion, and other findings. The generalized technique for segmentation is described in detail by Akhbardeh (2012) and was previously applied to the topic of breast MRI segmentation [21]. CLU uses the lung ultrasound video series to generate a single image, termed the “integrated image”, which highlights findings within the ultrasound video. Potentially pathological tissue is color-coded, ranging from cyan to orange-red, with normal tissue and background in dark blue. Ultrasound findings were also verified by radiologists with expertise in identifying lung ultrasound findings associated with COVID-19 and other pulmonary disorders.
2. Analysis of Orientation and Shape: This step extracts orientation and shape features that include the following: area, bounding-box, circulatory, convex area major and minor axis length, and orientation [25,26].
3. Decision-Making: The clinically significant findings and segmentations are retained—A-lines, B lines, pleural irregularity and effusion, consolidation, etc. using both the “integrated image” (the step 1 computer vision component that generates a single image) and the geometric/shape features (step 2). This step quantifies each finding: A-lines, B-lines, consolidation, and pleural effusion.
4. Calculated Lung Ultrasound Score: The final step calculates the “CLU Score”, with a normalized range from 0–100, by integrating the aforementioned shape and statistical features.

### 2.4. Performance Evaluation

Performance of the CLU was used to determine the presence or absence of the following findings: A-lines, patchy B-lines, confluent B-lines, thickened/irregular pleural lines, pleural effusion, subpleural consolidations, and consolidations with air bronchogram. The concordance of these findings was evaluated with board-certified clinical radiologists who are experts in pulmonary ultrasound serving as the gold standard. The results were recorded from the patient reports and used in the testing of the CLU index; they were verified by a board-certified radiologist.

### 2.5. Example Patient with Longitudinal Monitoring of COVID-19

We were able to obtain a patient that underwent longitudinal monitoring for COVID-19. A 35-year-old male patient was followed over a 20-day hospital course at La Paz University Hospital in Madrid, Spain [27]. After a diagnosis of COVID-19 via RT-PCR, the patient’s lungs were imaged via ultrasound each day for 20 days after a COVID-19 diagnosis. On days 1, 5, 10, and 20 after the COVID-19 diagnosis, CLU was applied to the ultrasound imaging, and the CLU score was calculated and compared to the reports.

## 3. Results

### 3.1. Comparison of CLU with Different Pathologies

**Figure 3:**
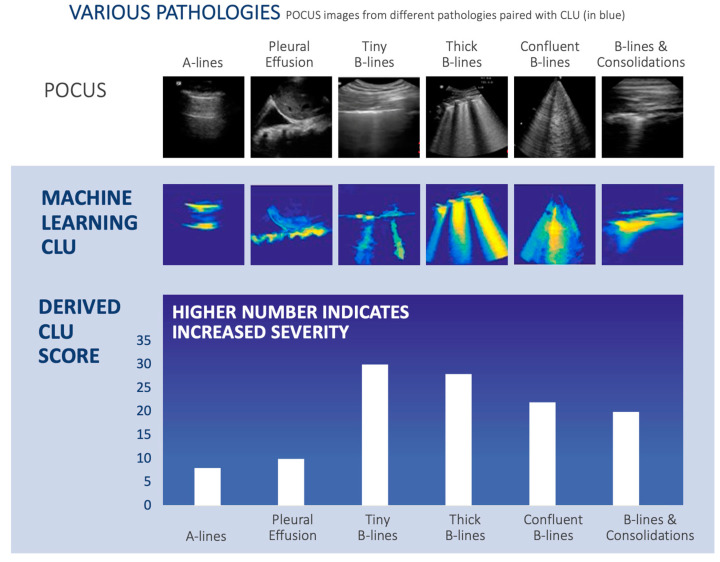
The ML-based POCUS CLU score. (**Top row**) Lung POCUS images in different pathologies in the “POCUS” row, with characteristic findings highlighted under the “machine leaning” row. Associated findings are color-coded, ranging from cyan to orange-red, with normal tissue and background in dark blue. (**Bottom row**) CLU score derived from POCUS. A normal-appearing CLU is ~10, whereas other findings increase the score above 10. POCUS = point of care ultrasound, CLU = calculated lung ultrasound.

Out of the 52 ultrasound examinations, we found the typical CLU US findings as shown in Figure 3, which shows the CLU algorithm outputs for the following lung ultrasound findings: A-lines, tiny (narrow) B-lines, confluent B-lines, pleural effusion, thick B-lines, and B-lines with consolidations. These results are summarized in Table 1.

Importantly, the CLU areas that captured normal tissue resulted in a lower score closer to blue, while the CLU areas that imaged increased pulmonary disease burden resulted in a higher score in the orange-red range.

### 3.2. The Use of CLU for Monitoring Pulmonary Disease Burden Longitudinally

**Figure 4:**
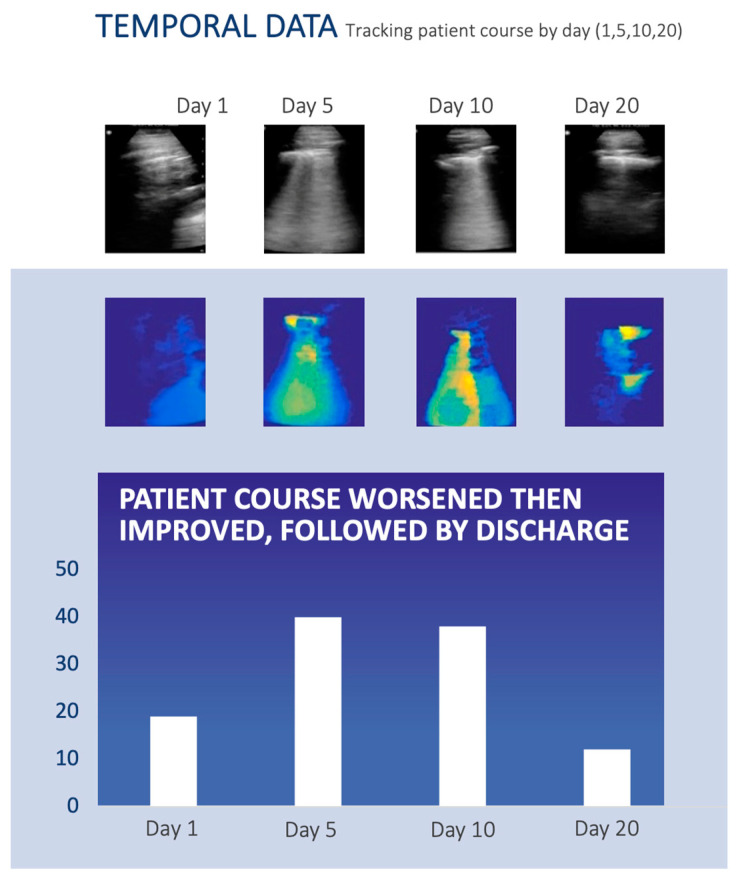
POCUS for monitoring COVID-19 over a 20-day hospital stay. Snapshot of frames from ultrasound videos from a COVID-19 case for days 1, 5, 10, and 20 after a polymerase chain reaction (PCR) positive test. After day 19, a negative PCR result was obtained. (**Top and middle rows**) Middle frames from POCUS with the CLU “integrated image” that highlights findings from POCUS including A/B-lines and consolidations (normal in dark blue, abnormal in orange to red). (**Bottom row**) The CLU metric after day 1: confluent/focal B-lines on days 5 and 10, consolidation on day 5, and thick pleural lines from days 10 to 20.

Figure 4 demonstrates the longitudinal use of CLU from days 1, 5, 10, and 20 for a 35-year-old male after being diagnosed as positive for COVID-19. The top row shows that on day one, there were no significant lung POCUS abnormalities. After five days, COVID-19 had progressed within the lungs to include typical findings of B-7 lines (B-lines ≥ 7 mm apart) confluent with subpleural consolidations bilaterally in the posterior lower lobes. By day 10, there were extensive thick pleural lines and focal B-lines. Finally, by day 20, there was significant improvement with remaining thickened pleura and B-lines. The patient had a negative COVID-19 PCR test on day 20.

### 3.3. Comparison of Performance between CLU and Radiologists

The imaging findings using CLU demonstrated excellent concordance with radiologists for all findings (Table 1). These included A-lines (12), patchy B-lines (19), confluent B-lines (17), thickened/irregular pleural lines (13), pleural effusion (6), subpleural consolidations (12), and consolidations with air bronchogram (9). For each finding in each row of Table 1, CLU demonstrated concordance in identifying the relevant radiological findings in the 52-ultrasound examination dataset.

## 4. Discussion

We have developed and tested the CLU method on an existing cohort of COVID-19 ultrasound data, with excellent performance in identifying the pulmonary patterns associated with COVID-19.

With further clinical validation, this tool has the potential to save radiologists time and increase their efficiency in reading studies, by quantifying findings and creating preliminary reports, pre-populated with findings of interest that the radiologist can quickly verify. CLU was able to detect areas of interest in COVID-19 and quantify relevant findings, such as A/B-lines, consolidation, pleural effusion, etc. There are several lung ultrasound findings associated with COVID-19, and ultrasound has been shown to be of utility due to its safety, lack of radiation, low cost, repeatability, and use in point-of-care settings [28]. This technique does not rely upon a particular ultrasound device, operating system, or hardware configuration. Hence, it can be rapidly scaled up and applied to any ultrasound scanner worldwide, from traditional devices to point-of-care scanners attached to smartphones.

However, not all sonographers and physicians have significant prior experience with lung ultrasound for COVID-19, especially in resource-limited regions with a dearth of trained sonographers and radiologists. For instance, healthcare providers in the developing world, including physicians, nurses, and technicians, have identified a lack of training as a primary barrier to the use of ultrasound in their practice [29]. Ultrasound evaluation is also operator dependent. Detecting A- or B-lines on ultrasound examinations is not always straightforward, and there is a learning curve for radiologists to become sufficiently familiar with performing lung ultrasound.

Both traditional machine learning and deep learning have previously been applied to analyzing lung ultrasound images for COVID-19. Wang et al. analyzed 27 patients using features such as B-lines and pleural lines in conjunction with a support vector machine to classify patients as severe or non-severe, achieving an area under the curve (AUC) of 0.96 [30]. Diaz-Escobar et al. adapted pre-trained deep learning architectures (VGG19, InceptionV3, Xception, ResNet50) on 3326 pulmonary ultrasound frames from the POCUS dataset; InceptionV3 performed best, achieving an AUC of 0.971 for distinguishing COVID-19 from bacterial pneumonia and healthy lungs [31]. Mento et al. used a standardized imaging protocol for lung ultrasound in COVID and applied it to 314,879 frames from 1488 lung ultrasound videos in 82 patients; they evaluated performance on a video level by aggregating frame-based scores from deep learning, and the agreement between deep learning and lung ultrasound experts for the stratification of patients as high versus low risk for clinical worsening was 86.0% [32].

Interestingly, there may be issues with analyzing individual frames rather than patients. Roshankhah et al. analyzed 1863 B-mode images from 203 videos [33]. Signs of lung damage were manually segmented, and lungs were scored on a 0–3 scale. They used a U-Net neural network and performed a simple 90:10 percent train-test split either at the individual image (or frame) level or at the patient level (ensuring that the same patient does not have frames in both the training and testing tests). While the accuracy at the image level was 95%, the accuracy at the patient level was lower at 63–73% under different scenarios. It is essential that any frame-based analyses in the literature establish the training and testing sets appropriately, as otherwise algorithmic performance can be falsely elevated. In addition, the manual segmentation involved in training several models presented in the literature could be time-consuming and tedious, particularly for a large dataset. Both issues are avoided in our method.

Finally, other techniques for COVID lung ultrasound have been performed. For instance, Barros et al. combined a convolutional neural network (CNN) with a long short-term memory (LSTM) component to learn the temporal dependence of the data [34]. This hybrid CNN-LSTM had an average accuracy of 93% and sensitivity of 97% for COVID-19, outperforming purely spatial models. Horry et al. created a multimodal dataset that combined X-ray, CT, and ultrasound to address potential issues related to having limited data on a particular modality [35]. They used publicly available data and well-established deep learning models in conjunction with transfer learning. Using ultrasound, they achieved a sensitivity of 97% and a positive predictive value of 99% for classifying COVID-19 and pneumonia versus normal. Karnes et al. used few-shot learning to distinguish between healthy controls, pneumonia, and COVID-19, with satisfactory initial results using small dataset sizes [36]. Additional studies using variations of the aforementioned techniques have been described [37].

The CLU method has several features that render it novel: (1) it is software-only and does not require specific ultrasound hardware, facilitating its use on any platform, including PC, smartphone, and tablet; (2) it employs a patient-specific, unsupervised learning model that does not require training on a large dataset; (3) it can quantify disease burden by establishing the presence or absence of key ultrasound findings and monitor them through the course of clinical treatment. This patient-specific approach, compared to conventional artificial intelligence and machine learning/deep learning, does not need to be trained, as it is fully unsupervised. This obviates the need for largescale datasets for training an algorithm, although we do plan future studies to clinically validate the algorithm’s results.

Compared to X-ray and CT, applications of AI to ultrasound have been comparatively fewer. Ultrasound presents unique challenges for applying AI, including operator dependence and differences in image acquisition techniques. X-ray and CT, on the other hand, consist of either 2D or 3D images collected using a more uniform imaging protocol. Medical imaging startup Buttery Network, the creator of the Butterfly iQ portable ultrasound device, has collected data from portable ultrasounds performed using its device via a cloud-based system, and it believes that as it obtains more data, its image analytics toolset will progressively become more accurate [38]. However, this analysis focuses on one ultrasound manufacturer, detailed information regarding its performance is not available, and image analytic techniques are typically more robust if they work across different platforms and scanner imaging techniques, a key strength of our technique.

This technique has worldwide utility, including individuals with a COVID-19 diagnosis who may require longitudinal monitoring of treatment response. Although the pandemic has continued for a few years, the emergence of variants such as delta and omicron, the large number of individuals who refuse vaccination, and continued outbreaks in places with limited vaccines or poor-quality vaccines result in many potential patients who may need to be evaluated for COVID-19. This technique, used in conjunction with POCUS, could be used to monitor patients for treatment response, quantitatively assessing pulmonary disease burden and informing healthcare workers regarding the extent of pulmonary findings within the lungs. Due to the inexpensive nature of ultrasound, this software can be deployed in hospitals worldwide, from top academic institutions to resource-limited regions, such as rural India and Africa.

Even now, when the acute phase of the COVID-19 pandemic is over, this technology could be adapted to other diseases, such as pneumonia and COPD exacerbation. For instance, a recent meta-analysis showed that lung ultrasound could diagnose pneumonia with high accuracy (AUC = 0.95), outperforming chest radiography and correlating highly with chest CT, which involves an ionizing radiation beam [39]. In addition, both pneumonia and COPD exacerbation are part of the BLUE-Protocol, a decision tree that incorporates findings from the lung ultrasound exam [6]. Pneumonia and COPD have significant global morbidity and mortality. A total of 212 million cases of COPD were reported in 2019, with 3.3 million deaths and 74.4 million disability-adjusted life years [40]. According to the World Health Organization, pneumonia accounted for 14% of all deaths in children under five, resulting in 740,180 deaths in 2019 [41]. We also anticipate that these techniques could be extended to other pulmonary diseases in the future [42,43].

With respect to the CLU score, Figure 3 showed that there was a general increase in this score in pathological disease states compared to normal (A-lines), and Figure 4 shows one patient example in which the CLU score generally worsened and then improved with the disease state. However, further clinical validation and fine-tuning are needed for this scoring aspect of the algorithm. For instance, in Figure 3, the score for pleural effusion was higher than that for A-lines, but it was not substantially higher, as were the other findings. Also, it will be necessary to have more longitudinal cases that track pulmonary disease severity. Nevertheless, this does not obviate the algorithm’s success in identifying characteristic lung ultrasound findings in this patient cohort.

Regarding future research, a logical next step would be to apply this technique to publicly available datasets that have proliferated over the past few years, which contain hundreds of ultrasound examinations with video [37]. We also plan further clinical validation using a large amount of prospective patient data. This could include examining the concordance between the lung ultrasound findings determined by radiologists and the CLU score [10]. It could also include having the same sonographer image the same patient twice on the same day to assess intra-observer variability, as well as having two different sonographers image the same patient to assess inter-observer variability. While having a diverse and heterogenous dataset (with different scanners and patient populations) was important for the development of this algorithm for the purposes of model generalizability, for future clinical validation, especially for use at any particular clinical site, it will be essential that there is a homogeneous scanning method with optimal settings that mirrors the high-level clinical practice at that site in order to have the most robust clinical performance metrics [44].

To test the robustness of the CLU technique in low-resource settings, we will assess the CLU’s performance with low-resolution and low-framerate video, including video captured from a cellphone camera. We will also consider extending CLU to other diseases, such as tuberculosis, interstitial lung disease, or congestive heart failure, or diseases involving specific patient populations, such as neonatal respiratory distress syndrome. In addition, future directions include extending this analysis to patients with long-COVID, residual clinical symptoms long after the initial disease episode; these patients will have a longstanding need to monitor pulmonary disease burden. Lung ultrasound has shown utility for the follow-up of these patients [45]. Although COVID-19 may be better controlled in the future, new variants, vaccine resistance, and distribution issues in developing nations can complicate the situation. As a result, there is utility in adapting CLU to quantitatively assess pulmonary disease burden for other illnesses.

## 5. Conclusions

This preliminary study demonstrated excellent concordance between the CLU technique and radiologist readings for seven lung ultrasound findings, noting that future validation is needed on a larger dataset. This concordance between the radiologist and CLU serves as a promising starting point, suggesting that with future development this technique could be of utility when making care decisions about patients with respiratory illness, facilitating early and proper intervention.

## 6. Patents

A.A. and M.A.J. have the patent, “Multiparametric non-linear dimension reduction methods and systems related thereto”, US Patent 9,256,966 [46]. A.A. under Ambient Digital LLC has a patent application for CLU.

## Figures and Tables

**Table 1:** Imaging findings on lung ultrasound for 52 ultrasound examinations, as well as CLU concordance with the radiologist report.

Finding	CLU Finding	Radiologist Finding
A-Lines	12	12
Patchy B-lines	19	19
Confluent B-lines	17	17
Thickened, Irregular Pleural Lines	13	13
Pleural Effusion	6	6
Subpleural Consolidations	12	12
Consolidations with Air Bronchogram	9	9

## Data Availability

Data will be made available upon request, and availability is determined by the institutional guidelines.

## References

[1] Coronavirus Resourse Center, Johns Hopkins University COVID-19 Dashboard by the Center for Systems Science and Engineering (CSSE) at Johns Hopkins University (JHU). https://coronavirus.jhu.edu/map.html.

[2] Salehi S., Abedi A., Balakrishnan S., Gholamrezanezhad A. (2020). Coronavirus Disease 2019 (COVID-19): A Systematic Review of Imaging Findings in 919 Patients. Am. J. Roentgenol..

[3] Jacobi A., Chung M., Bernheim A., Eber C. (2020). Portable chest X-ray in coronavirus disease-19 (COVID-19): A pictorial review. Clin. Imaging.

[4] Mossa-Basha M., Meltzer C.C., Kim D.C., Tuite M.J., Kolli K.P., Tan B.S. (2020). Radiology Department Preparedness for COVID-19: Radiology Scientific Expert Review Panel. Radiology.

[5] Mutambudzi M., Niedwiedz C., Macdonald E.B., Leyland A., Mair F., Anderson J., Celis-Morales C., Cleland J., Forbes J., Gill J. (2020). Occupation and risk of severe COVID-19: Prospective cohort study of 120 075 UK Biobank participants. Occup. Environ. Med..

[6] Lichtenstein D.A. (2015). BLUE-protocol and FALLS-protocol: Two applications of lung ultrasound in the critically ill. Chest.

[7] Sultan L.R., Sehgal C.M. (2020). A Review of Early Experience in Lung Ultrasound in the Diagnosis and Management of COVID-19. Ultrasound Med. Biol..

[8] European Society of Radiology (ESR) (2019). ESR statement on portable ultrasound devices. Insights Imaging.

[9] Poggiali E., Dacrema A., Bastoni D., Tinelli V., Demichele E., Mateo Ramos P., Marciano T., Silva M., Vercelli A., Magnacavallo A. (2020). Can Lung US Help Critical Care Clinicians in the Early Diagnosis of Novel Coronavirus (COVID-19) Pneumonia?. Radiology.

[10] Manivel V., Lesnewski A., Shamim S., Carbonatto G., Govindan T. (2020). CLUE: COVID-19 lung ultrasound in emergency department. Emerg. Med. Australas..

[11] Dargent A., Chatelain E., Kreitmann L., Quenot J.P., Cour M., Argaud L., COVID-LUS study group (2020). Lung ultrasound score to monitor COVID-19 pneumonia progression in patients with ARDS. PLoS ONE.

[12] Soldati G., Smargiassi A., Inchingolo R., Buonsenso D., Perrone T., Briganti D.F., Perlini S., Torri E., Mariani A., Mossolani E.E. (2020). Proposal for International Standardization of the Use of Lung Ultrasound for Patients With COVID-19: A Simple, Quantitative, Reproducible Method. J. Ultrasound Med..

[13] Deng Q., Zhang Y., Wang H., Chen L., Yang Z., Peng Z., Liu Y., Feng C., Huang X., Jiang N. (2020). Semiquantitative lung ultrasound scores in the evaluation and follow-up of critically ill patients with COVID-19: A single-center study. Acad. Radiol..

[14] Lichter Y., Topilsky Y., Taieb P., Banai A., Hochstadt A., Merdler I., Gal Oz A., Vine J., Goren O., Cohen B. (2020). Lung ultrasound predicts clinical course and outcomes in COVID-19 patients. Intensive Care Med..

[15] Buonsenso D., Piano A., Raffaelli F., Bonadia N., de Gaetano Donati K., Franceschi F. (2020). Point-of-Care Lung Ultrasound findings in novel coronavirus disease-19 pnemoniae: A case report and potential applications during COVID-19 outbreak. Eur. Rev. Med. Pharmacol. Sci..

[16] Cohen P., Gebo K., Post T.W. (2023). COVID-19: Management of adults with acute illness in the outpatient setting. UpToDate.

[17] Saraogi A. (2015). Lung ultrasound: Present and future. Lung India.

[18] Kopinski H., Davis L. A—Lines—Normal Lung. http://www.thepocusatlas.com/lung/5l9jgyaszu0othj5tidg0miqxkmvyv.

[19] Taylor T., Meer J., Beck S. (2015). Emergency Ultrasound: Lung Assessment. Lung ultrasound takes 2 to 3 minutes to perform and can help narrow down the differential in a patient with dyspnea. Emerg. Med..

[20] Buda N., Cylwik J., Mroz K., Rudzinska R., Dubik P., Malczewska A., Oraczewska A., Skoczynski S., Suska A., Gorecki T. (2021). Lung Ultrasound Examination in Patients with SARS-CoV-2 Infection: Multicenter Study. J. Clin. Med..

[21] Akhbardeh A., Jacobs M.A. (2012). Comparative analysis of nonlinear dimensionality reduction techniques for breast MRI segmentation. Med. Phys..

[22] Otsu N. (1979). A Threshold Selection Method from Gray-Level Histograms. IEEE Trans. Syst. Man Cybern..

[23] Arthur D., Vassilvitskii S. K-Means++: The Advantages of Careful Seeding. Proceedings of the SODA 2007: Proceedings of the Eighteenth Annual ACM-SIAM Symposium on Discrete Algorithms.

[24] Meyer F. (1994). Topographic distance and watershed lines. Signal Process..

[25] Lehmann G., Legland D. Efficient N-Dimensional Surface Estimation Using Crofton Formula and Run-Length Encoding. https://insight-journal.org/browse/publication/852.

[26] Shoemake K., Heckbert P.S. (1994). Graphics Gems IV.

[27] Macias M., Riscinti M. Ultrasound in COVID-19. http://www.thepocusatlas.com/covid19.

[28] Peng Q.Y., Wang X.T., Zhang L.N., Chinese Critical Care Ultrasound Study Group (2020). Findings of lung ultrasonography of novel corona virus pneumonia during the 2019–2020 epidemic. Intensive Care Med..

[29] Shah S., Bellows B.A., Adedipe A.A., Totten J.E., Backlund B.H., Sajed D. (2015). Perceived barriers in the use of ultrasound in developing countries. Crit. Ultrasound J..

[30] Wang Y., Zhang Y., He Q., Liao H., Luo J. (2022). Quantitative Analysis of Pleural Line and B-Lines in Lung Ultrasound Images for Severity Assessment of COVID-19 Pneumonia. IEEE Trans. Ultrason. Ferroelectr. Freq. Control.

[31] Diaz-Escobar J., Ordonez-Guillen N.E., Villarreal-Reyes S., Galaviz-Mosqueda A., Kober V., Rivera-Rodriguez R., Lozano Rizk J.E. (2021). Deep-learning based detection of COVID-19 using lung ultrasound imagery. PLoS ONE.

[32] Mento F., Perrone T., Fiengo A., Smargiassi A., Inchingolo R., Soldati G., Demi L. (2021). Deep learning applied to lung ultrasound videos for scoring COVID-19 patients: A multicenter study. J. Acoust. Soc. Am..

[33] Roshankhah R., Karbalaeisadegh Y., Greer H., Mento F., Soldati G., Smargiassi A., Inchingolo R., Torri E., Perrone T., Aylward S. (2021). Investigating training-test data splitting strategies for automated segmentation and scoring of COVID-19 lung ultrasound images. J. Acoust. Soc. Am..

[34] Barros B., Lacerda P., Albuquerque C., Conci A. (2021). Pulmonary COVID-19: Learning Spatiotemporal Features Combining CNN and LSTM Networks for Lung Ultrasound Video Classification. Sensors.

[35] Horry M.J., Chakraborty S., Paul M., Ulhaq A., Pradhan B., Saha M., Shukla N. (2020). COVID-19 Detection Through Transfer Learning Using Multimodal Imaging Data. IEEE Access.

[36] Karnes M., Perera S., Adhikari S., Yilmaz A. Adaptive Few-Shot Learning PoC Ultrasound COVID-19 Diagnostic System. Proceedings of the 2021 IEEE Biomedical Circuits and Systems Conference (BioCAS).

[37] Wang J., Yang X., Zhou B., Sohn J.J., Zhou J., Jacob J.T., Higgins K.A., Bradley J.D., Liu T. (2022). Review of Machine Learning in Lung Ultrasound in COVID-19 Pandemic. J. Imaging.

[38] Hunter T. Diagnostics, Monitoring, Drug Discovery: How AI Is Fighting COVID-19. https://builtin.com/artificial-intelligence/ai-coronavirus.

[39] Kameda T., Mizuma Y., Taniguchi H., Fujita M., Taniguchi N. (2021). Point-of-care lung ultrasound for the assessment of pneumonia: A narrative review in the COVID-19 era. J. Med. Ultrason..

[40] Safiri S., Carson-Chahhoud K., Noori M., Nejadghaderi S.A., Sullman M.J.M., Ahmadian Heris J., Ansarin K., Mansournia M.A., Collins G.S., Kolahi A.A. (2022). Burden of chronic obstructive pulmonary disease and its attributable risk factors in 204 countries and territories, 1990–2019: Results from the Global Burden of Disease Study 2019. BMJ.

[41] WHO Pneumonia in Children. https://www.who.int/news-room/fact-sheets/detail/pneumonia.

[42] Giannelli F., Cozzi D., Cavigli E., Campolmi I., Rinaldi F., Giache S., Rogasi P.G., Miele V., Bartolucci M. (2022). Lung ultrasound (LUS) in pulmonary tuberculosis: Correlation with chest CT and X-ray findings. J. Ultrasound.

[43] Xie H.Q., Zhang W.W., Sun S., Chen X.M., Yuan S.F., Gong Z.H., Liu L. (2019). A simplified lung ultrasound for the diagnosis of interstitial lung disease in connective tissue disease: A meta-analysis. Arthritis Res. Ther..

[44] Allen B., Dreyer K., Stibolt R., Agarwal S., Coombs L., Treml C., Elkholy M., Brink L., Wald C. (2021). Evaluation and Real-World Performance Monitoring of Artificial Intelligence Models in Clinical Practice: Try It, Buy It, Check It. J. Am. Coll. Radiol..

[45] Giovannetti G., De Michele L., De Ceglie M., Pierucci P., Mirabile A., Vita M., Palmieri V.O., Carpagnano G.E., Scardapane A., D’Agostino C. (2021). Lung ultrasonography for long-term follow-up of COVID-19 survivors compared to chest CT scan. Respir. Med..

[46] Jacobs M.A., Akhbardeh A. (2016). Multiparametric Non-Linear Dimension Reduction Methods and Systems Related Thereto. U.S. Patent.

